# Functional head impulse test and video head impulse test in interictal Ménière’s disease and inferior vestibular neuritis: a preliminary comparative study

**DOI:** 10.3389/fneur.2026.1851411

**Published:** 2026-07-13

**Authors:** Fumiyuki Goto, Yukiko Tsuda, Kenji Okami, Koichiro Wasano

**Affiliations:** 1 Department of Otolaryngology-Head and Neck Surgery, Tokai University School of Medicine, Isehara, Kanagawa, Japan; 2 Goto ENT & Dizziness Clinic, Machida, Tokyo, Japan

**Keywords:** caloric-vHIT dissociation, dynamic visual acuity (DVA), functional head impulse test (fHIT), inferior vestibular neuritis, interictal period, Ménière’s disease, video head impulse test (vHIT)

## Abstract

**Background and objective:**

In interictal Ménière’s disease (MD), a caloric-vHIT dissociation is well established: caloric testing reveals vestibular hypofunction, yet video head impulse test (vHIT) gains in the lateral canal are preserved. Whether this preservation corresponds to preserved functional gaze stabilization remains unknown. This preliminary, exploratory study compared affected and unaffected ear responses of dynamic visual acuity (DVA), functional head impulse test (FHIT), and vHIT in interictal MD, and evaluated the clinical value of each measure.

**Methods:**

Eleven interictal MD patients (10 female, 1 male; mean age 61.6 ± 16.7 years) and 12 inferior vestibular neuritis (IVN) patients (6 female, 6 male; mean age 61.5 ± 9.9 years) were enrolled. IVN was defined by reduced posterior canal vHIT gain (<0.7) or absent cVEMP on the affected side. All tests were performed during the interictal phase (≥48 h after the last episode). Six-canal FHIT (correct answer rate, CA%), six-canal vHIT, and DVA were measured. Lesion-side versus intact-side comparisons used the Wilcoxon signed-rank test.

**Results:**

In MD, lesion-side FHIT-H CA% was significantly lower than intact-side (53.9 ± 33.5% vs. 77.3 ± 26.5%; *p* = 0.022), whereas vHIT-H gain (*p* = 0.146) and DVA (*p* = 0.820) showed no significant laterality. The FHIT laterality index (25.4 ± 31.4%) significantly exceeded the vHIT-H laterality index (4.2 ± 9.1%; *p* = 0.019; *r* = 0.71); the FHIT-H abnormality rate was 8/11 (73%) versus vHIT-H 2/11 (18%). In IVN, lesion-side posterior canal vHIT gain was significantly reduced (*p* = 0.027), while FHIT-H showed no laterality (*p* = 0.413).

**Conclusion:**

MD and IVN showed contrasting patterns: MD exhibited selective lesion-side FHIT-H reduction with preserved vHIT, whereas IVN showed selective posterior canal vHIT reduction with normal FHIT-H laterality. These findings suggest that FHIT may reflect endolymphatic hydrops-related functional vestibular changes distinct from the structural nerve lesions characteristic of IVN. FHIT may be a useful adjunct for detecting functional vestibular asymmetry in interictal MD; these findings are hypothesis-generating and require replication in larger prospective cohorts.

## 1 Introduction

Ménière’s disease (MD) is an inner ear disorder characterized clinically by recurrent episodes of rotatory vertigo, fluctuating sensorineural hearing loss, tinnitus, and aural fullness. Endolymphatic hydrops is a common pathological finding associated with MD. In vestibular assessment of MD, caloric testing frequently reveals hypofunction on the affected side, whereas video head impulse test (vHIT) gains in the horizontal canal are maintained within the normal range during the interictal period. This caloric-vHIT dissociation is now recognized as a hallmark of MD (1–3).

Video head impulse test quantifies vestibulo-ocular reflex (VOR) gain in response to high-acceleration head impulses and has high sensitivity for diagnosing acute unilateral vestibular loss. However, it has been suggested that VOR gain does not necessarily correspond to preservation of dynamic visual acuity (DVA) in daily life (4). The functional head impulse test (FHIT, also termed fvHIT) was recently developed to assess not only VOR gain but also the integrated function of corrective saccades and the visual system—referred to as “functional gaze stabilization ability”—by measuring the correct answer rate (CA%) of an optotype presented during head impulses (5). FHIT has been shown to detect functional deficits even when vHIT is normal (6), and has also been applied as an outcome measure for vestibular rehabilitation (7).

Dynamic visual acuity is a task in which subjects identify optotypes while the head is oscillated sinusoidally, primarily reflecting VOR function in the low-to-mid frequency range. Regarding the application of FHIT and DVA to MD, Balayeva et al. (2023) (8) reported that FHIT CA% was lower in both MD and vestibular migraine patients than in healthy controls; however, that study did not examine the lesion-side vs. intact-side difference in FHIT. To our knowledge, no previous report has compared FHIT asymmetry between the affected and unaffected sides in interictal MD.

The present study used DVA, FHIT, and vHIT to compare functional vestibular function between the affected and unaffected sides in interictal MD. Patients with inferior vestibular neuritis (IVN) were included as a control group. Given the retrospective and observational nature of this study, we did not test a formal hypothesis; rather, we descriptively compared the lesion-side sensitivity of FHIT and vHIT and explored possible pathophysiological correlates.

## 2 Materials and methods

### 2.1 Participants

Patients who visited Goto ENT & Dizziness Clinic (Machida, Tokyo) between October 2025 and March 2026 were enrolled. Twelve IVN cases were included as a control group. All tests were performed during the interictal phase (at least 48 h after the last vertiginous episode). The mean interval between the last vertiginous episode and testing was 18.4 ± 14.2 days (range 3–52) in the MD group and 31.6 ± 22.8 days (range 7–89) from symptom onset in the IVN group.

MD group: Eleven patients with definite MD fulfilling the Bárány Society (2015) diagnostic criteria (9) were enrolled (10 female, 1 male; mean age 61.6 ± 16.7 years; right-affected n = 4, left-affected n = 7).

IVN group: Twelve patients were included as controls. Diagnostic criteria for IVN were based on dysfunction of inferior vestibular nerve-innervated structures (posterior semicircular canal and saccule): at least one of the following was required—(1) reduced posterior canal vHIT gain (<0.7) on the affected side, or (2) absent cVEMP on the affected side (10, 11). The anterior canal was excluded from the diagnostic criteria because it is innervated by the superior vestibular nerve. To exclude superior vestibular nerve (SVN) involvement, anterior-canal vHIT gain (a marker of SVN function) was assessed in all IVN patients and was preserved within the normal range in the majority of cases (mean 0.86 ± 0.29; 78% ≥ 0.7). Preserved oVEMP responses on the affected side provided additional supportive evidence. Together, these findings indicate that the dysfunction was largely confined to the inferior vestibular nerve territory without significant SVN involvement. Of 14 candidates, 2 with normal posterior canal vHIT and normal cVEMP were excluded, yielding 12 IVN patients (posterior canal gain reduction only n = 3, absent cVEMP only *n* = 2, both findings *n* = 7; 6 female, 6 male; mean age 61.5 ± 9.9 years; right-affected *n* = 7, left-affected *n* = 5).

This was a retrospective observational study. Patient information was managed using anonymized case numbers. This study was reviewed and approved by the Ethics Committee of the Association for the Promotion of Ethical Review in Clinical Research (Tokyo, Japan) (Approval No. E2026-02-001; approval date: February 2, 2026). Given the retrospective nature of the study, informed consent was waived by the ethics committee.

### 2.2 Dynamic visual acuity (DVA)

DVA was measured using the Interacoustics VisualEyes system. Patients oscillated their head in the horizontal (right, left) and vertical (up, down) directions at a constant velocity while identifying the orientation of a Landolt C optotype displayed on a monitor. DVA was recorded as the difference from static visual acuity (logMAR); larger values indicate greater dynamic visual acuity impairment. Head oscillations were performed at an initial speed of 150°/s, frequency of approximately 2 Hz, and amplitude of approximately ±15°. Ten trials were performed per direction, and the mean value was used for analysis. A metronome was used to maintain constant motion speed.

### 2.3 Functional head impulse test (FHIT)

Functional head impulse test was performed using the Interacoustics VisualEyes system (fvHIT module). Passive head impulses were delivered in six canal planes (horizontal: rightward and leftward; LARP: right-posterior and left-anterior; RALP: right-anterior and left-posterior), and the CA% of a Landolt C optotype presented at the peak of each impulse was recorded. Head impulses were delivered with peak velocities of 150–250°/s and peak accelerations of approximately 1,500–3,000°/s^2^. A minimum of 20 valid impulses per canal direction was obtained (mean 24 ± 4). The Landolt C optotype was presented at the velocity peak of each impulse with its gap orientation randomized among four directions, and the response window was 80 ms. Impulses with peak velocity <100°/s or duration >200 ms were automatically rejected by the system as invalid. The lower limit of normal CA% was set at 80%, with values below 80% defined as abnormal, based on previously published data (5, 6).

### 2.4 Video head impulse test

Video head impulse test was performed using the Impulse system (Otometrics) to measure VOR gain across all six semicircular canals. Normal range was defined as ≥0.8 for the horizontal canal and ≥0.7 for the vertical canals. Air-conducted cVEMP was used to assess saccular/inferior vestibular nerve function. Ocular VEMP (oVEMP) was additionally obtained as a supplementary measure of utricular/superior vestibular nerve (SVN) function to help exclude SVN involvement. An absent response was regarded as abnormal.

### 2.5 Lesion-side classification

The lesion side was determined using a predefined hierarchy: (1) pure-tone audiometry (primary criterion in MD: low-frequency sensorineural hearing loss ≥10 dB relative to the contralateral ear); (2) imaging findings (MRI-FLAIR endolymphatic hydrops signal, when available); and (3) clinical history (side of vertigo, aural fullness, and tinnitus). When all three modalities were available (MD n = 8, IVN n = 9), inter-modality agreement on the lesion side was 100%; in the remaining cases, audiometry and clinical history were concordant.

### 2.6 Statistical analysis

Throughout this study, the suffix “-H” denotes the horizontal canal, “-P” the posterior canal, and “-A” the anterior canal (e.g., FHIT-H, horizontal-canal FHIT; vHIT-H, horizontal-canal vHIT; vHIT-P, posterior-canal vHIT). Lesion-side vs. intact-side comparisons were performed using the Wilcoxon signed-rank test. Detection rates of FHIT and vHIT abnormalities and laterality indices ([intact-side value − lesion-side value] / [intact-side value + lesion-side value] × 100) were calculated. Between-group comparisons used the Mann–Whitney U test. Statistical analyses were performed using GraphPad Prism 11 (GraphPad Software, San Diego, CA, USA). Statistical significance was set at *p* < 0.05. Effect sizes for Wilcoxon signed-rank tests were calculated as r = Z/√N and interpreted as small (≥0.1), medium (≥0.3), or large (≥0.5). Ninety-five percent confidence intervals (CIs) for proportions were calculated using the Wilson score method. Given the multiple canal comparisons, a Bonferroni-corrected threshold (*α* = 0.008 for six canal directions) was additionally considered for the exploratory secondary analyses; the primary hypothesis (lesion-side FHIT-H reduction in MD) was pre-specified. *Post hoc* power was estimated for the principal comparisons. As this was an exploratory pilot study, the analyses are intended to be hypothesis-generating.

## 3 Results

### 3.1 Participant characteristics

**Figure 1:**
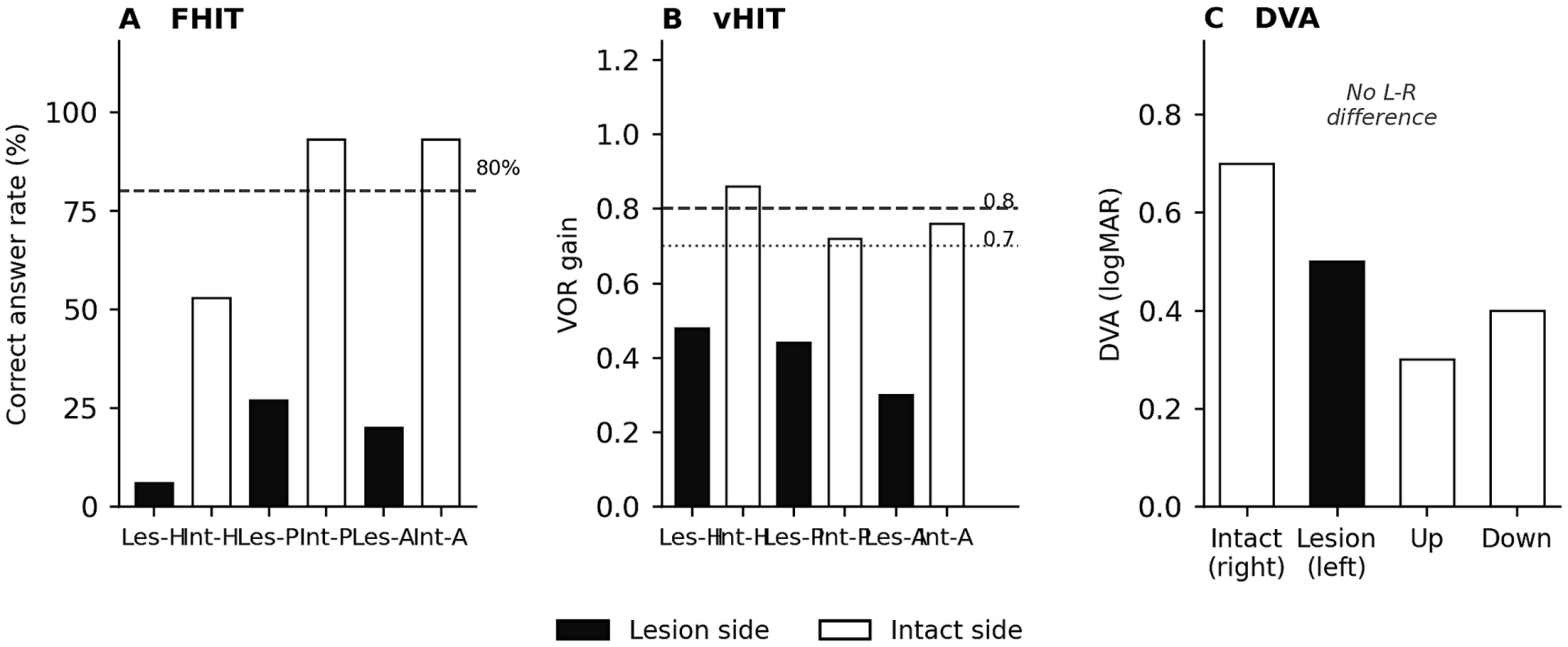
Representative case: interictal left-sided Ménière’s disease. **(A)** FHIT correct answer rate (CA%) for six semicircular canals. The lesion-side horizontal canal (Les-H) shows markedly reduced CA% (6%), well below the 80% cutoff (dashed line), while intact-side and vertical canals are relatively preserved. **(B)** vHIT VOR gain. The lesion-side horizontal canal (0.48) falls below the 0.8 cutoff, and vertical canals also show reduced gain. **(C)** DVA (logMAR) shows no lesion–intact difference. Les = Lesion side; Int = Intact side; H = Horizontal; P = Posterior; A = Anterior.

MD group (*n* = 11): mean age 61.6 ± 16.7 years (range 39–85), 10 female and 1 male. IVN group (*n* = 12): mean age 61.5 ± 9.9 years (range 50–81), 6 female and 6 male. Affected side in MD: right *n* = 4, left *n* = 7; in IVN: right *n* = 7, left *n* = 5. No significant age difference was found between groups (*p* = 0.975). All tests were performed during the interictal phase. Representative case FHIT, vHIT, and DVA findings are shown in Figure 1.

### 3.2 Lesion-side vs. intact-side comparisons

**Figure 2:**
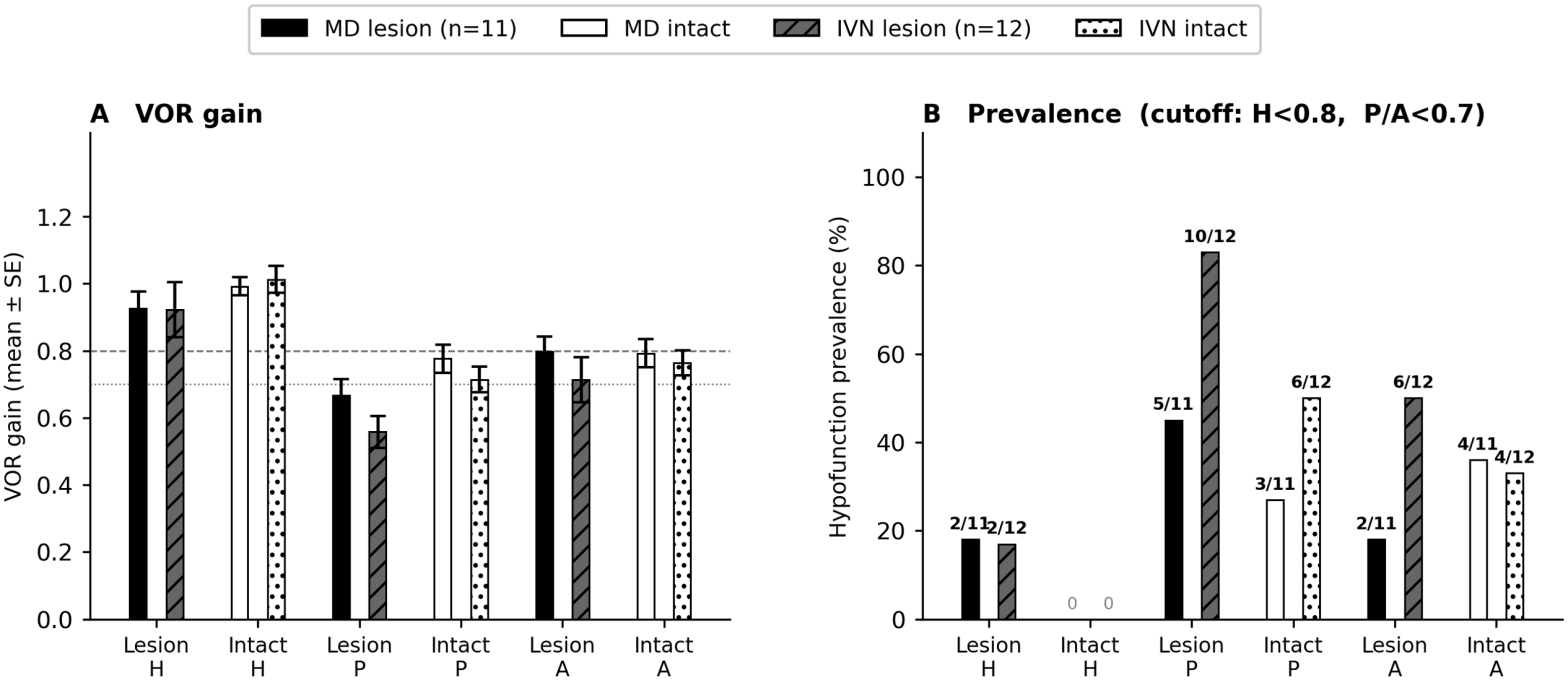
Lesion vs. intact side—MD (*n* = 11, **A–D**) and IVN (*n* = 12, **E–H**). **(A–D)** MD group (*n* = 11, upper row); **(E–H)** IVN group (*n* = 12, lower row). Columns: **(A,E)** = FHIT H-canal correct answer rate (CA%); **(B,F)** = FHIT P-canal CA%; **(C,G)** = vHIT H-canal VOR gain; **(D,H)** = vHIT P-canal VOR gain. Filled circles = lesion side; open circles = intact side; bars = mean ± SD; dashed lines = cutoff (80% for FHIT; 0.8 for vHIT H-canal; 0.7 for vHIT P-canal). Brackets with *p*-values: Wilcoxon signed-rank test (lesion vs. intact). †: MD vs. IVN Mann–Whitney *U* test (lesion side).

In the MD group, FHIT-H CA% was significantly lower on the lesion side than the intact side (53.9 ± 33.5% vs. 77.3 ± 26.5%; *p* = 0.022; *r* = 0.69, large effect). No significant differences were found for vHIT-H gain (lesion 0.925 ± 0.17 vs. intact 0.992 ± 0.089; *p* = 0.146), vHIT-P gain (lesion 0.666 ± 0.164 vs. intact 0.776 ± 0.14; *p* = 0.084), or DVA (lesion 0.355 ± 0.23 vs. intact 0.327 ± 0.276 logMAR; *p* = 0.820). In the IVN group, vHIT-P gain was significantly lower on the lesion side (0.558 ± 0.163 vs. 0.714 ± 0.132; *p* = 0.027; *r* = 0.64, large effect). No significant differences were found for FHIT-H (lesion 55.7 ± 30.4% vs. intact 67.1 ± 19.5%; *p* = 0.413) or vHIT-H (lesion 0.922 ± 0.281 vs. intact 1.012 ± 0.140; *p* = 0.308) (Figure 2).

### 3.3 Between-group comparison (MD vs. IVN)

Between-group comparisons revealed no statistically significant differences in lesion-side FHIT-H CA% (53.9 ± 33.5% vs. 55.7 ± 30.4%; *p* > 0.99), lesion-side vHIT gain (0.925 ± 0.17 vs. 0.922 ± 0.281; *p* = 0.175), or FHIT laterality index (25.4 ± 31.4% vs. 13.1 ± 39.3%; *p* = 0.406). However, contrasting within-group patterns were observed: MD showed significant lesion-side FHIT-H reduction (*p* = 0.022) while IVN showed no FHIT-H laterality (*p* = 0.413), whereas IVN showed significant lesion-side vHIT-P reduction (*p* = 0.027) while MD showed no significant vHIT-P laterality (*p* = 0.084) (Figure 2).

### 3.4 Detection rates and laterality indices

FHIT-H abnormality on the lesion side (CA% < 80%) was detected in 8/11 cases (72%) in the MD group, whereas vHIT-H abnormality (<0.8) was detected in only 2/11 (18%). The FHIT laterality index (25.4 ± 31.4%) was significantly larger than the vHIT-H laterality index (4.2 ± 9.1%; *p* = 0.019), demonstrating that FHIT more clearly reflects lesion-side asymmetry than vHIT.

### 3.5 Canal-specific vHIT abnormality patterns

**Figure 3:**
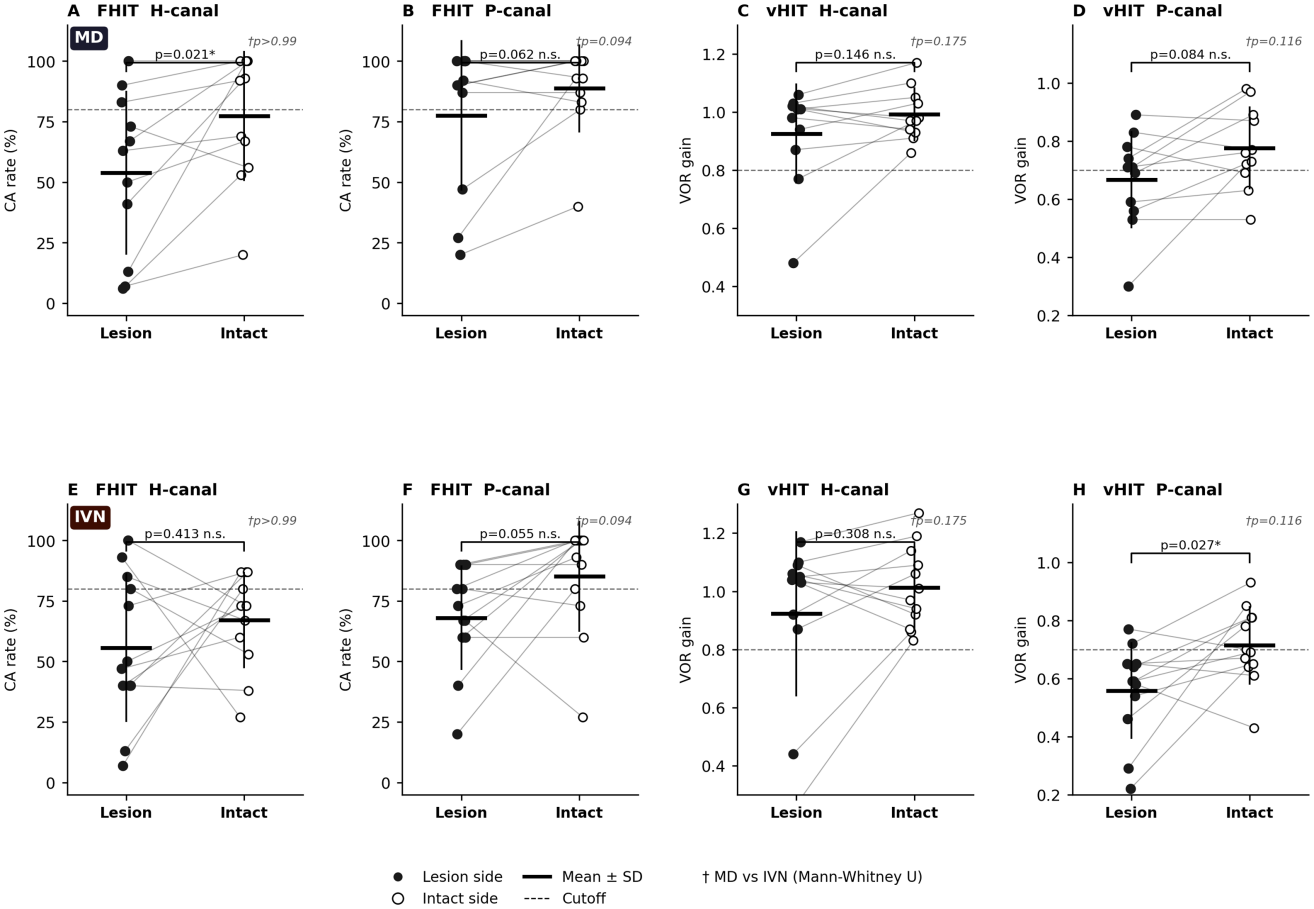
Canal-specific vHIT analysis: VOR gain and hypofunction prevalence in MD (*n* = 11) vs. IVN (*n* = 12). **(A)** VOR gain (mean ± SE) for lesion and intact sides of each semicircular canal (H = Horizontal, P = Posterior, A = Anterior). Dashed line = 0.8 cutoff (H-canal); dotted line = 0.7 cutoff (P- and A-canals). **(B)** Prevalence of hypofunction per canal (%). Numbers above bars indicate affected/total cases. Black-filled bars = MD lesion side; white bars = MD intact side; gray-hatched bars = IVN lesion side; dotted-white bars = IVN intact side.

In the MD group, lesion-side horizontal canal gain reduction (<0.8) was limited to 2/11 cases (18%), while posterior canal (lesion-side 45%, intact-side 27%) and anterior canal (lesion-side 45%, intact-side 36%) showed higher rates of reduction. In the IVN group, posterior canal abnormality rate was highest at 9/12 (75%), in marked contrast to MD (27%), while horizontal canal abnormality was low at 1/12 (8%). This contrasting pattern is considered to reflect differences in the pathophysiology of MD and IVN (Figure 3).

## 4 Discussion

### 4.1 Dissociation between normal vHIT and lesion-side FHIT reduction

The most important finding of this study is the dissociation observed in interictal MD: despite near-normal vHIT horizontal canal gains bilaterally, FHIT-H was significantly reduced on the lesion side. This dissociation suggests that recovery of VOR gain does not necessarily correspond to recovery of dynamic visual-cognitive function.

Video head impulse test primarily measures VOR responses to high-acceleration head impulses (3,000–6,000°/s^2^), mainly reflecting the function of irregularly discharging vestibular afferents. In contrast, FHIT applies an optotype identification task during the same head impulses, providing a comprehensive assessment of “functional gaze stabilization ability” that integrates VOR function, corrective saccades, and visual processing. In MD, intermittent fluctuation of vestibular input due to endolymphatic hydrops may persist during the interictal period, with continued variability in afferent neural activity. Such subtle intermittent vestibular fluctuations may be difficult to capture as VOR gain changes, but can be sensitively detected by functional tests combining cognitive tasks such as FHIT.

Balayeva et al. (2023) (8) reported lower FHIT CA% in MD patients compared to healthy controls, but did not perform lesion-side vs. intact-side comparisons. The present study adds novelty by demonstrating, for the first time, selective FHIT reduction on the lesion side in interictal MD.

### 4.2 Why DVA shows no lesion-intact difference

DVA is performed while oscillating the head at a constant velocity, obtained during predictable, sinusoidal head oscillations. A key mechanistic distinction between DVA and FHIT lies not simply in stimulus frequency but in the predictability of the head motion. During DVA, the regular oscillatory motion allows the central nervous system to preprogram compensatory eye movements and recruit predictive (efference-copy) mechanisms, which can mask a mild functional deficit. In contrast, FHIT employs unpredictable, high-acceleration head impulses that require real-time visuovestibular integration without the benefit of predictive compensation. This difference in predictability—rather than frequency per se—may explain why FHIT detected lesion-side dysfunction in interictal MD whereas DVA did not. The high-acceleration, unpredictable nature of FHIT may therefore make it more suitable for detecting subtle functional vestibular asymmetry in MD.

### 4.3 Canal-specific vHIT abnormality patterns

In the 11 MD cases, lesion-side horizontal canal gain reduction was limited to 2 cases (18%), while posterior and anterior canal abnormalities (lesion-side 45% each) predominated. In contrast, the IVN group showed the highest posterior canal abnormality rate at 75%, with only 8% horizontal canal abnormality. The distribution of vestibular dysfunction observed in MD may be consistent with preferential involvement of the inferior vestibular nerve, which supplies the saccule and lies in anatomical proximity to the cochlear blood supply (12). The concurrent involvement of the anterior canal (superior vestibular nerve innervation) and posterior canal (posterior-inferior vestibular nerve innervation) suggests that endolymphatic hydrops extends beyond the otolith organs to multiple vestibular nerve pathways.

Furthermore, the preservation of horizontal canal vHIT in all MD cases is consistent with the hypothesis proposed by McGarvie et al. (2015) (13) that endolymphatic hydrops-induced dilation of the membranous semicircular canal selectively affects responses to low-frequency caloric stimulation, thereby forming the caloric-vHIT dissociation in MD. The longer preservation of horizontal canal function relative to the vertical canals may reflect differences in the distribution of endolymphatic expansion.

### 4.4 Clinical applications of FHIT

The results of this study suggest that FHIT may serve as an auxiliary tool for lesion-side identification in interictal MD. Caloric testing is difficult to repeat in daily clinical practice due to its cumbersome procedure and patient burden, and MRI-based endolymphatic hydrops imaging is limited to specialized facilities. FHIT can evaluate all six semicircular canals non-invasively within approximately 5–10 min, and the FHIT laterality index may provide supplementary information for lesion-side identification. Furthermore, FHIT holds potential as an outcome measure for vestibular rehabilitation.

### 4.5 Limitations

This study is a single-center retrospective case series with a limited sample size (MD *n* = 11, IVN *n* = 12). Comparisons that did not reach statistical significance require revalidation with larger cohorts. The interval between the last vertiginous episode and testing was not standardized, and the relationship between episode frequency and FHIT performance remains a topic for future investigation. For the IVN group, construction of a more homogeneous cohort using stricter diagnostic criteria would be desirable. The use of absent cVEMP as a criterion for IVN assumes saccular dysfunction relatively specific to the inferior vestibular nerve; although recent studies have questioned the absolute specificity of cVEMP, we mitigated this concern primarily by confirming preserved anterior-canal vHIT, supported by preserved oVEMP responses, thereby excluding significant superior vestibular nerve involvement. Nonetheless, some residual cohort heterogeneity cannot be fully excluded. In addition, the interval between the last vertiginous episode and testing was not standardized (MD: mean 18.4 ± 14.2 days, range 3–52; IVN: mean 31.6 ± 22.8 days, range 7–89), which may have introduced variability given the fluctuating nature of interictal MD. The MD group was also predominantly female (10/11), reflecting the known epidemiology of MD; although the FHIT optotype task is not expected to show clinically meaningful sex differences, this imbalance is acknowledged. Because IVN is defined primarily by posterior canal dysfunction, its preserved horizontal FHIT-H is partly expected from anatomy; accordingly, the principal contribution of this study is the within-group demonstration of lesion-side FHIT-H reduction in MD despite preserved vHIT, rather than the between-group contrast itself. Finally, *post hoc* power for the principal comparisons was approximately 60–65%, and the primary FHIT-H finding (*p* = 0.022) did not survive Bonferroni correction (*α* = 0.008); these results should therefore be regarded as exploratory and hypothesis-generating. The objective of robustly establishing the diagnostic value of FHIT in interictal MD should ultimately be addressed by a prospective study with a well-designed methodology, an adequate sample size, sufficient statistical power, and a healthy control group; the present retrospective observations are intended to provide preliminary support and rationale for such future work.

## 5 Conclusion

In interictal MD, vHIT-H gain showed no significant lesion-intact side difference (*p* = 0.146). FHIT demonstrated significant reduction on the lesion side in the FHIT-H direction (lesion-side 53.9% vs. intact-side 77.3%; *p* = 0.022), sensitively capturing functional vestibular dysfunction undetectable by vHIT. The FHIT-H lesion-side abnormality rate (72%) far exceeded that of vHIT-H (18%), and the FHIT laterality index showed significantly greater lesion-intact asymmetry compared to the vHIT-H laterality index (25.4% vs. 4.2%; *p* = 0.019). In the IVN group, vHIT-P was significantly reduced on the lesion side (*p* = 0.027), with a posterior canal abnormality rate reaching 83%. In this preliminary exploratory study, FHIT appears to be a potentially useful adjunct measure for detecting functional vestibular asymmetry in interictal MD. These findings are hypothesis-generating and require confirmation in larger prospective cohorts, ideally including a healthy control group, before clinical recommendations can be made.

## Data Availability

The raw data supporting the conclusions of this article will be made available by the authors, without undue reservation.
